# Using Machine Learning for Predicting the Best Outcomes With Electrical Muscle Stimulation for Tremors in Parkinson’s Disease

**DOI:** 10.3389/fnagi.2021.727654

**Published:** 2021-09-10

**Authors:** Onanong Phokaewvarangkul, Peerapon Vateekul, Itsara Wichakam, Chanawat Anan, Roongroj Bhidayasiri

**Affiliations:** ^1^Department of Medicine, Faculty of Medicine, Chulalongkorn Centre of Excellence for Parkinson’s Disease and Related Disorders, Chulalongkorn University and King Chulalongkorn Memorial Hospital, Bangkok, Thailand; ^2^Department of Computer Engineering, Faculty of Engineering, Chulalongkorn University, Bangkok, Thailand; ^3^The Academy of Science, The Royal Society of Thailand, Bangkok, Thailand

**Keywords:** Parkinson’s disease, electrical muscle stimulation, machine learning, tremor, Parkinson’s glove, resetting mechanism

## Abstract

Recent studies have identified that peripheral stimulation in Parkinson’s disease (PD) is effective in tremor reduction, indicating that a peripheral feedback loop plays an important role in the tremor reset mechanism. This was an open-label, quasi-experimental, pre- and post-test design, single-blind, single-group study involving 20 tremor-dominant PD patients. The objective of this study is to explore the effect of electrical muscle stimulation (EMS) as an adjunctive treatment for resting tremor during “on” period and to identify the best machine learning model to predict the suitable stimulation level that will yield the longest period of tremor reduction or tremor reset time. In this study, we used a Parkinson’s glove to evaluate, stimulate, and quantify the tremors of PD patients. This adjustable glove incorporates a 3-axis gyroscope to measure tremor signals and an EMS to provide an on-demand muscle stimulation to suppress tremors. Machine learning models were applied to identify the suitable pulse amplitude (stimulation level) in five classes that led to the longest tremor reset time. The study was registered at the www.clinicaltrials.gov under the name “The Study of Rest Tremor Suppression by Using Electrical Muscle Stimulation” (NCT02370108). Twenty tremor-dominant PD patients were recruited. After applying an average pulse amplitude of 6.25 (SD 2.84) mA and stimulation period of 440.7 (SD 560.82) seconds, the total time of tremor reduction, or tremor reset time, was 329.90 (SD 340.91) seconds. A significant reduction in tremor parameters during stimulation was demonstrated by a reduction of Unified Parkinson’s Disease Rating Scale (UPDRS) scores, and objectively, with a reduction of gyroscopic data (*p* < 0.05, each). None of the subjects reported any serious adverse events. We also compared gyroscopic data with five machine learning techniques: Logistic Regression, Random Forest, Support Vector Machine (SVM), Neural Network (NN), and Long-Short-Term-Memory (LSTM). The machine learning model that gave the highest accuracy was LSTM, which obtained: accuracy = 0.865 and macro-F1 = 0.736. This study confirms the efficacy of EMS in the reduction of resting tremors in PD. LSTM was identified as the most effective model for predicting pulse amplitude that would elicit the longest tremor reset time. Our study provides further insight on the tremor reset mechanism in PD.

## Introduction

Tremor is found in 70% of patients with Parkinson’s disease (PD), usually occurring at rest (Hughes et al., 1993; Deuschl et al., 1998). Resting tremors can cause considerable disability for an individual with PD (Hughes et al., 1993), often leading to stigmatization, feelings of shame, and psychological difficulties (Zimmermann et al., 1994; Simpson et al., 2013). The standard treatment of parkinsonian tremors are dopaminergic and anticholinergic medications, but the outcomes are often unreliable when compared to treatment outcomes for other cardinal motor symptoms, such as bradykinesia and rigidity (Jimenez and Vingerhoets, 2012). Moreover, there are no evidence-based therapeutic guidelines that provide the efficacy of specific dopaminergic medications for parkinsonian tremors (Ferreira et al., 2013; Connolly and Lang, 2014). Current therapeutic options for patients who fail to respond adequately to standard treatment include surgical interventions or infusion therapies, which can be associated with various side effects (Benabid et al., 1996; Koller et al., 1997; Krack et al., 1998a,b; Limousin et al., 1998; Pinter et al., 1999; Marjama-Lyons and Koller, 2000; Pogarell et al., 2002; Crosby et al., 2003; Katzenschlager et al., 2003; Ferreira et al., 2013). Therefore, there is a real need to identify a new efficacious treatment, with fewer adverse events, for this symptom.

Although not exactly known, the mechanism of tremorogenesis in PD is complex, generated by interactions between central and peripheral (local mechanical-reflex) loops (Hallett, 1998; Deuschl et al., 2000; McAuley and Marsden, 2000; Deuschl et al., 2001) with the contralateral primary motor cortex likely to be the major driver for tremor in activities selected by the basal ganglia (putamen, globus pallidus, and subthalamic nucleus) (Helmich et al., 2012; Hirschmann et al., 2013). The amplitude of tremor is probably determined by a network within the cerebellum acting through the thalamus (Helmich et al., 2011). The role of peripheral mechanisms for tremor in PD is less clear, but is considered to be involved in the maintenance and modulation of the amplitude of tremor (Deuschl et al., 2000; McAuley and Marsden, 2000). While central tremor generation is the main target for most current PD treatments (e.g., thalamotomy or thalamic DBS), some evidence has indicated that peripheral stimulation involving either mechanical resonance (such as bone, joint, and soft tissue) or feedback resonance (represented as a reflex mechanism) can also generate or modulate tremor even when driven from a central origin (McAuley and Marsden, 2000). In one early study, supramaximal electrical peripheral nerve stimulation inhibited rhythmic EMG activity in parkinsonian tremor for a period of around 200 ms with the duration of inhibition varying inversely with ongoing tremor (Britton et al., 1993). Another study also demonstrated a significant attenuation of parkinsonian tremor (up to 62%) following electrical muscle stimulation (EMS) of the afferent muscles (Javidan et al., 1992). As muscles can generate tremor, direct and strong mechanical conditions imposed upon the muscle might be able to reset tremor, even if that tremor originates from a central source.

Based on promising results from early studies (Mones and Weiss, 1969; Bathien et al., 1980; Lee and Stein, 1981; Britton et al., 1992, 1993), recent investigators have explored the potential of EMS as a treatment of parkinsonian tremor. While efficacy has been replicated in a number of recent studies, others have reported conflicting results associated with potential side effects (Javidan et al., 1992; Maneski et al., 2011; Zhang et al., 2011; Gallego et al., 2013; Bo et al., 2014; Dosen et al., 2014). Our group recently explored the benefits of EMS in the reduction of medically refractory resting tremor in PD patients in both open-label and randomized-controlled studies (Jitkritsadakul et al., 2015, 2017). Significant reductions in transient tremor were achieved during EMS which correlated with a reduction in the clinical tremor score obtained from the Unified Parkinson’s Disease Rating Scale (UPDRS) (Jitkritsadakul et al., 2015, 2017). This preliminary evidence supported the possibility of a tremor reset mechanism from EMS (Jitkritsadakul et al., 2015). From direct visual observation, following a 10-s stimulation period, tremor reduction remained stable for a further 10 s after withdrawal of EMS before returning to baseline levels (Jitkritsadakul et al., 2015). Using a 30-s square-wave stimulation, some patients had visual tremor reductions for up to 3 min before their tremor re-emerged to the pre-stimulation level—we interpreted this period as a tremor reset time (Jitkritsadakul et al., 2017). It seems that a longer period of stimulation may produce a longer period of tremor reduction (Jitkritsadakul et al., 2015, 2017).

Recent advances in machine learning analytics in PD have allowed the development of methods that can recognize human motion using training information obtained from multiple sensors. Deep learning, as a type of machine learning that typically involves multilayered neural networks to perform a variety of input-output modeling tasks, has been shown to be capable of detecting tremor (Yao et al., 2019), motor fluctuations (Aich et al., 2020), and freezing of gait (Sigcha et al., 2020) in PD; however, no prior studies have investigated the role of machine learning for predicting the best outcomes with EMS for tremor in PD. We hypothesized that EMS had a direct tremor reduction effect, and the higher pulse amplitude and/or the greater stimulation time may produce a longer tremor reset time. Therefore, the objectives of this study were to: (1) evaluate the effect of EMS and the tremor reduction in PD; (2) assess the level of pulse amplitude that produces significant tremor reduction with the longest tremor reset time; and (3) identify the best machine-learning model for predicting stimulation levels that yield the longest tremor reset time.

## Materials and Methods

This was an open-label, quasi-experimental, pre- and post-test design, single-blind, single-group study involving 20 PD patients. The study was conducted at Chulalongkorn Centre of Excellence for Parkinson’s Disease and Related Disorders^1^ The study protocol was approved by the Institutional Review Board, Faculty of Medicine, Chulalongkorn University (IRB 483/57). The IRB granted us to permission this treatment as an adjunctive treatment during “on” period when patients were optimized on oral medications. Information regarding this research study was provided and written informed consent was obtained from every subject at enrollment in accordance with the declaration of Helsinki. The study was registered at the www.clinicaltrials.gov under the name “The Study of Rest Tremor Suppression by Using Electrical Muscle Stimulation” (NCT02370108) with the translation of study protocol provided as the Supplementary Material 1. Supplementary Figure 1 provides a complete detailed CONSORT flowchart. A TREND checklist is provided as the Supplementary Material 2. All subjects met the clinical diagnosis criteria for PD according to the United Kingdom Parkinson’s Disease Society Brain Bank criteria (Hughes et al., 1993). As tremors in Parkinson’s disease patients are heterogeneous presentation and classified in 3 different types of tremor patterns, including class I tremor, class II tremor, and class III tremor. In order to reduce clinical heterogeneity of tremor, we recruited only PD patients with type 1, classic Parkinsonian tremor according to the consensus statement of the Movement Disorder Society (Deuschl et al., 1998). In addition, all patients must have drug resistant tremor as defined as the subjects either failed to experience a clinically relevant and useful improvement in tremor under an optimized antiparkinsonian therapy with various agents, or side effects encountered under an effective anti-tremor therapy were intolerable. We also recruited only subjects whose tremor were unresponsive to at least 2 oral dopaminergic medications and anticholinergics (Jitkritsadakul et al., 2015). None of participants received device-aided therapies, such as infusion therapy or deep brain stimulation. Similar details of inclusion and exclusion criteria were shown in our previous study (Jitkritsadakul et al., 2015). The calculation of the sample size was based on the results of the RMS of the angular velocity before and during stimulation from previous publication (Jitkritsadakul et al., 2015), which suggested that at least 7 subjects were required for the comparison for tremor outcomes in this study (Supplementary Material 3).

### Procedures

Before enrollment, all recruited patients received stable anti-parkinsonian medications for at least 30 days. A movement disorder neurologist (OP) conducted individual interviews with participants to obtain their demographics and clinical data relevant to the study. Parkinsonian and tremor assessments, both clinical and objective, were done for each patient to determine the severity of PD symptoms during both “off” and “on” periods. Physical examinations of each patient were recorded by video for later review. Parkinsonian symptoms were clinically assessed according to the UPDRS and Hoehn and Yahr (HY) scale. Marked tremor criteria are clinically defined by a sum score of at least 8 out of 32 on UPDRS tremor items 16, 20, and 21 (tremor score), or, if the tremor is present on one side only, by a tremor score of at least 6 out of 32 (Zimmermann et al., 1994; Pogarell et al., 2002). Parkinsonian tremors were objectively assessed with the tremor detection module of a Parkinson’s glove during both “off” and “on” periods, however, the assessment of tremor during stimulation was done during “on” periods only (2–3 h after the last intake of medications) in order to evaluate the additional benefits of EMS for tremor reduction. Standard oral medications for PD tremor usually give a maximum of 50% tremor reduction for each dose (Kartzinel et al., 1976; Pogarell et al., 2002), therefore, in this study, tests during the “on” period were only conducted to see whether EMS could provide additional benefits to tremor reduction as an adjunctive treatment to current oral medications.

### Parkinson’s Glove

**FIGURE 1:**
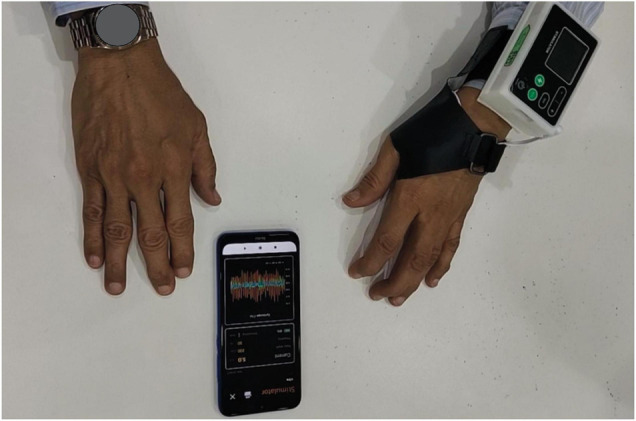
The Parkinson’s glove. The Parkinson’s glove is a specially designed adjustable glove, which incorporates a tremor detection module and an electrical muscle stimulation module to detect and suppress resting hand tremors. The control panel was developed using an Android application on a mobile phone connected via Bluetooth to the Parkinson’s glove.

In this study, we used the Parkinson’s glove to evaluate, stimulate, and quantify parkinsonian tremors. A Parkinson’s glove is a unique adjustable glove used to treat tremors, which integrates both a tremor detection module and an EMS module, and is worn on the most tremulous hand (Figure 1). The tremor detection module was a 3-axis gyroscope that measures tremor signals from angular displacements of motion (Gallego et al., 2010). A fast Fourier transform algorithm was calculated and graphed for tremor amplitude and frequency. The EMS module was designed to function identically to the approved EMS standard, and received approval for electronic product testing from the electrical and electronic products testing center (PTEC) in Thailand. The EMS module provided continuous stimulation with a square-wave pulse to the affected muscles via two surface electrodes under either manual or automatic control. The controller, with an embedded sensor, was placed over the patient’s wrist. The control panel was developed using an Android application on a mobile phone connected via a Bluetooth connection to the Parkinson’s glove. Real-time tremor signals and stimulation protocols were transferred automatically and recorded in the internal memory of a mobile phone, which could then be uploaded onto a Cloud system for further analysis. Similar details for the Parkinson’s glove development are shown in our previous study (Jitkritsadakul et al., 2017). A patent application number of the Parkinson’s glove has been filed to the Thai Intellectual Property Office (Application number 1701000170).

### Tremor Experiment

**FIGURE 2:**
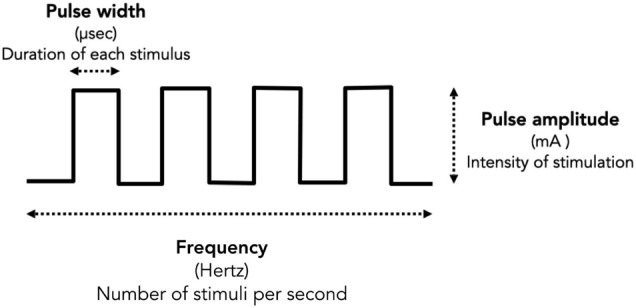
Stimulation parameters. There are main three stimulation parameters determine for the size and intensity of each stimulus of an EMS including pulse amplitude, pulse width, and frequency. Pulse amplitude is the intensity of the stimulation, measured in milliampere (mA). Pulse width is the duration of each stimulus, measured in microseconds. Frequency is the number of stimuli delivered each second, measured in hertz (Hz).

**FIGURE 3:**
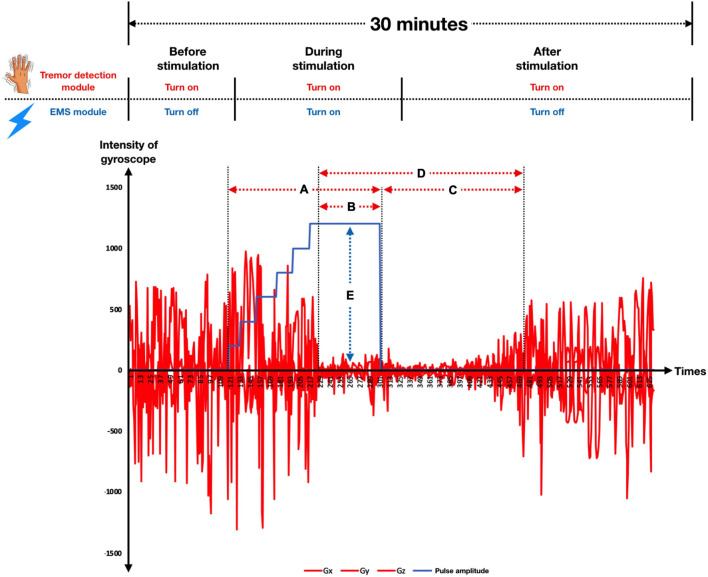
The temporal pattern of the experiment is illustrated, including the period of tremor detection and EMS, as well as tremor reduction outcomes. In this study, we divided the experiment into 3 sections, including (1) before stimulation, (2) during stimulation, and (3) after stimulation. The red line denotes the intensity of the gyroscope (x, y, and z), while the blue line refers to the pulse amplitude. Black dashed lines represent time boundaries. The duration of stimulation is indicated by **(A)**, while **(B)** shows the duration of tremor reduction during stimulation. The duration of continuing tremor reduction after withdrawal of EMS until the tremor re-emerged to the pre-stimulation tremor level is shown by **(C)**, while **(D)** indicates the tremor reset time and **(E)** represents Pulse amplitude.

Tremor assessment was conducted in a quiet room. All participants wore the Parkinson’s glove on their most tremulous hand with two self-adhesive electrodes placed over the thenar muscle as well as the first and second interosseous muscles. Participants were instructed to sit comfortably in an armchair and asked to close their eyes while counting backward to encourage the resting tremor. After turning on the Parkinson’s glove, the tremor detection module was used to quantify the intensity of tremor signals throughout the session. Meanwhile, an EMS module was used to stimulate the patient’s hand muscles to reduce tremors. Stimulation was performed manually by the attending physician to identify the most effective stimulation protocol for tremor reduction. There are three main stimulation parameters for an EMS: pulse amplitude, pulse width, and frequency. As depicted in Figure 2, pulse amplitude is the intensity of the stimulation, measured in milliampere (mA), pulse width is the duration of each stimulus, measured in microseconds (μs), and frequency is the number of stimuli delivered per second, measured in hertz (Hz). In this study, each subject only underwent the experimental procedure once, and the total experiment time was approximately 30 min for each subject. We divided the experiment into 3 sections as follows (Figure 3).

1. Before stimulation: The period when the tremor detection module is turned on, but the EMS module is still turned off. Tremor signals were measured continuously by the tremor detection module to give baseline data. This section usually took 5 min.
2. During stimulation: The period when the tremor detection module and the EMS module were both turned on. Two stimulation parameters, frequency (50 Hz) and pulse width (150 μs), were kept constant while the attending physician increased the stimulation intensity by slowly raising the pulse amplitude by 1 mA approximately every 10 s (1 mA, 2 mA, 3mA,…, etc.) until the tremor was reduced and the criteria for tremor reduction achieved. If a maximum pulse amplitude was identified, the physician then provided continuous stimulation at that pulse amplitude for approximately further 30 s before turning off the EMS. Criteria for significant tremor reduction in this study were determined by direct observation, including (1) a more than 50% tremor reduction from the clinical rating scale (UPDRS tremor score), and/or (2) a more than 50% tremor reduction identified by real-time gyroscope signals. For patients who did not meet these significant tremor reduction criteria, the physician turned off the stimulation if a visualized tetanic muscle contraction was achieved without pain. Tremor signals were measured continuously by the tremor detection module during the whole stimulation period. This section usually took 10–15 min.
3. After stimulation: The period when the EMS module was turned off, but the tremor detection module remained on. After turning off the EMS, tremor signals were measured continuously by the tremor detection module until the tremor signals returned to pre-stimulation levels. The duration of tremor reduction from EMS initiation until tremor re-emergence to pre-stimulation levels is termed the tremor reset time (Jitkritsadakul et al., 2015). This section usually took 10 min.

Safety profiles were assessed by recording the frequencies and severity of reported adverse events during stimulation and in a 1-month follow-up period. Any new symptoms or worsening of pre-existing symptoms was documented for each participant.

### Data Collecting Process

The control panel for the Parkinson’s glove was developed as an Android application for mobile phones using a Bluetooth connection so that the investigators could control and save tremor signals and stimulation parameters (Figure 1). Real-time tremor signals in every axis and stimulation protocol were saved to the internal memory of a mobile phone and automatically uploaded to a Cloud system as.csv files for further analysis. Raw motion signals from the gyroscope were calculated using the root mean square of angular velocity for each axis (RMS_x_, RMS_y_, RMS_z_) and tremor frequency for each axis. After receiving the collected data, regression analysis was used to determine the predictive factors for the longest tremor reset time, and a supervised machine learning approach used to identify the suitable stimulation levels that will yield the longest tremor reset time.

### Data Analysis

The baseline characteristics and tremor parameters were summarized using either means and standard deviations (SD), or frequencies and percentages as appropriate. The Wilcoxon–Mann–Whitney test was used to compare the efficacy of stimulation based upon the tremor parameters between the before-stimulation and during-stimulation measurements. The Chi-square test was used for categorical data. Correlation analysis was performed using Spearman’s correlation to determine the relationship between stimulation parameters and tremor reduction outcomes. The correlation coefficient (r) was interpreted as the strength of correlation as either weak, moderate, or high correlation (Taylor, 1990). Multiple linear regression analysis was carried out to predict the factors that lead to the longest tremor reset time. The multiple correlation coefficient is an index of how well a dependent variable can be predicted from a linear combination of independent variables. It ranges from 0 (zero multiple correlations) to 1 (perfect multiple correlations) and the value of *R*^2^ is the coefficient of determination. A *p* < 0.05 (two-tailed) was considered statistically significant. Statistical analysis for this study was based on the SPSS program version 23.0 software (SPSS Inc., Chicago, IL).

### Automatic Pulse Amplitude Prediction Models

Normally, physicians use manually controlled variation of stimulation to achieve an appropriate pulse amplitude setting for EMS, based on tremor intensity. However, as tremor intensity can fluctuate between individual PD patients, this method is impractical in a real-life scenario. To overcome this challenge, it is crucial to develop a machine learning model to automatically predict the suitable pulse amplitude for individual patients. In this study, the pulse amplitude level (target variable) is divided into five stimulation classes suggested by physicians as shown in Table 1, representing low to high stimulation levels.

**TABLE 1:** Categorized stimulation class into 5 classes based on pulse amplitude levels.

**Pulse amplitude level (mA)**	**Stimulation class (low to high)**
0	0
1–5	1
6–10	2
11–15	3
> = 16	4

*mA, milliampere; Greater stimulation class represents higher pulse amplitude level.*

Developments using standard machine learning require several steps, including data preprocessing, model construction, and model evaluation (Bizzego et al., 2019). The first step, data pre-processing, was the capture of raw motion signals from the gyroscope (G_x_, G_y_, G_z_) along with pulse amplitude (I_t_) and timestamp (t). Then, these gyroscope signals were aggregated using Root Mean Square (RMS) during an interval of 1 s resulting in RMS of each axis (RMS_x_, RMS_y_, RMS_z_), so each observation (row) represented a signal of 1 s interval using RMS, with the main outcome for prediction stimulation level, categorized into one of the five classes (low to high). We also investigated the effect of the stimulation level at the previous timestep (t−1) since stimulation adjustment is usually gradual; for example, the pulse amplitude level “3” is normally changed to either “2” or “4,” not suddenly jumped to “0” or “5.” Therefore, there were four inputs in total (RMS_x_, RMS_y_, RMS_z,_ I_t–1_). During the training phase, we applied a training strategy called “the teacher forcing algorithm” (Williams and Zipser, 1989) by supplying observed sequence values as inputs. In our case, we supplied the pulse amplitude level from the previous step, adjusted by physicians into the model. For the testing phase, the model was evaluated on the data collected from the physician’s process. Thus, it is a limitation of this study that the previous stimulation level used for the model was based on observed data, not from the model prediction so in the discussion section, we have provided a suggestion of how to deploy our model in real-life scenarios. Also, some data points needed to be removed due to manual pulse amplitude adjustments, e.g., no pulse amplitude assigned during the tremor period as shown in Figure 3. These data points are removed because they are bad examples for the model and can weaken model performance. In summary, the main outcome for prediction was stimulation level, which was categorized into five classes (low to high) as shown in Table 1. There were two feature sets; (1) only gyroscope signals and (2) gyroscope signals with the previous stimulation level.

**FIGURE 4:**
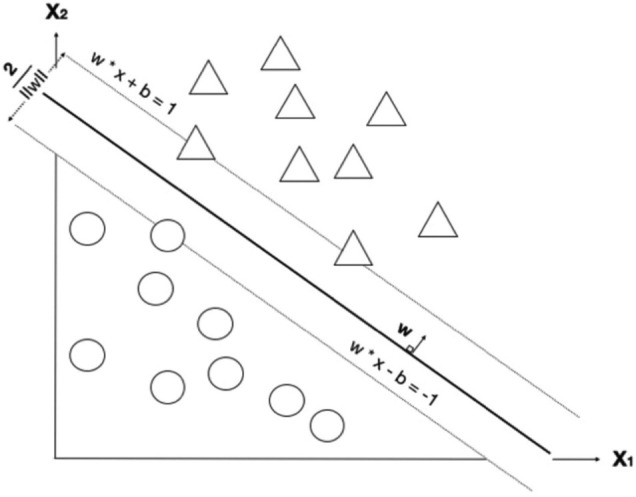
A decision boundary for support vector machine (SVM) that tries to maximize the boundary between two classes. Image shows a maximum-margin hyperplane and margin for an SVM trained on two classes. Samples on margins are called support vectors. x refers to each feature; there are two features in this example (x_1_ and x_2_). Each circle refers to a datum point, where blue and green refer to different classes. There are two learning parameters from the model; w and b are weights and bias, respectively. The margin between the two classes is 2/||w||.

The second development step concerns model construction. A machine learning model was applied to learn from the cleaned data using a multiclass classification that aimed to predict the pulse amplitude class (of the five) of stimulation required. Here we employed and compared five prediction techniques: (1) logistic regression, (2) random forest, (3) support vector machine (SVM) (Boser et al., 1992), (4) Neural Network (NN), and (5) long-short-term-memory (LSTM) (Hochreiter and Schmidhuber, 1997). Since this is a classification task, logistic regression was chosen as our baseline technique. Random forest is an ensemble of decision trees. It is developed to overcome the instability that a single classification or regression tree exhibits with minor perturbations of training data. Each tree is constructed using perturbed training data by sampling both rows (samples) and columns (variables); thus, it produces a variation among the trees in the ensemble. The settings for random forest were: the number of trees is 100, the number of sampled features is a squared root of total features, and observations are sampled using bootstrapping (sampling with replacement). SVM, one of more popular machine learning algorithms designed for a classification task, finds a separation hyperplane that maximizes between two classes, as shown in Figure 4 (Larhmam, 2018). The settings of SVM were: the kernel is radial basis function (RBF) and regularized parameter (C) is 1. Neural Network is built up with two hidden layers with 100 hidden units. Implementation of linear regression, random forest, SVM, and NN are all based on the scikit-learn library in Python.

**FIGURE 5:**
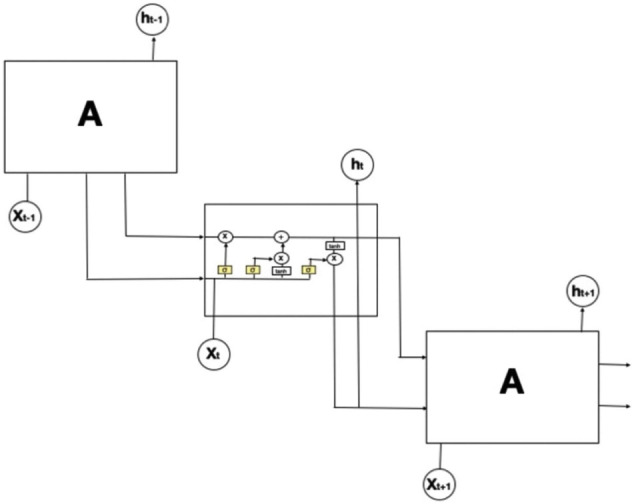
The structure of long-short-term-memory (LSTM). The repeating module in an LSTM contains four interacting layers. In this figure, an LSTM model is expanded into 3-time steps (t–1, t, t+1). The middle box refers to the model at the current time with an input x_t_. Since LSTM is a recurrent neural network, it also obtains two outputs from the previous time step: the lower arrow is the short-term memory output (h_t–1_) and the upper arrow is the long-term memory.

For the deep learning approach, LSTM was chosen since it is a recurrent neural network, commonly used for time–series data. It takes information from both the previous steps (short-term memory) and the long-term dependencies into account as shown in Figure 5 (Olah, 2015). The model is uni-directional, not bi-directional since we aim to predict the next time step; thus, future data cannot be used. The settings of LSTM were: there are two layers with 100 hidden units, follow by a dense layer with softmax activation and Adam optimization applied with the learning rate of 0.001. The implementation is based on the Tensorflow/Keras library.

**FIGURE 6:**
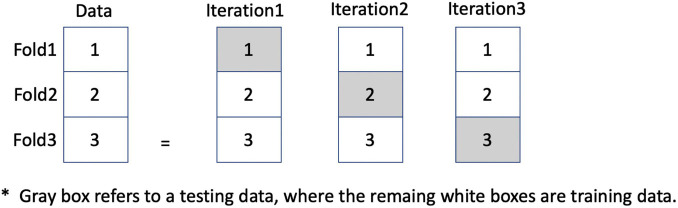
An illustration of threefold cross validation in order to avoid overfitting results. Gray box refers to testing data, where the remaining white boxes are training data.

The third, and final, step of model development is the evaluation. Here, all prediction models were applied and compared based on threefold cross validation as shown in Figure 6. Each fold contains a different set of patients, where the results are averaged on all three testing sets rather than just one set of testing data, so avoiding an overfitting result. In our data set, there were 20 PD patients, so there were 7, 7, and 6 patients in each fold, respectively. The data is separated into threefold by patients in order to avoid information leaking should data of the same patient be in both training and testing datasets. Table 2 illustrates the data statistics collected from 20 PD patients showing the number of records for each fold along with percentage of each stimulation class. However, each fold may have a slight difference in class distribution since the data is separated based on patients.

**TABLE 2:** Data statistics of data collected from 20 PD patients.

		**Class 0**	**Class 1**	**Class 2**	**Class 3**	**Class 4**	**Total**
Fold1	#records	3,191	1,280	573	406	87	5,537
7 PD’s	%records	57.16	25.60	9.39	6.45	1.38	100
Fold2	#records	1,798	1,879	1,076	228	0	4,981
7 PD’s	%records	34.68	43.33	18.54	3.44	0	100
Fold3	#records	3,303	775	2,558	796	9	7441
6 PD’s	%records	43.34	20.65	21.37	14.21	0.41	100
Total	#records	2,511	1,169	882	453	34	5,049
	%records	49.73%	23.15%	17.47%	8.97%	0.67%	100%

*Each row refers to an interval of 1 s. Only two patients with data in Class 4; thus, there are only twofolds with Class 4’s data.*

Evaluation measures, precision (P), recall (R or called sensitivity), and F1 (the harmonic mean between precision and recall) were calculated based on a confusion matrix that has True Positive (tp), True Negative (tn), False Positive (fp), and False Negative (fn) as the components. The calculation method of P, R, and F1 is shown in the equation below. Since there are five classes, the overall performance is an averaging of performance from all classes called “Macro Averaging” (Macro-Precision, Macro-Recall, and Macro-F1).


$$P=\frac{tp}{tp+fp},R=\frac{tp}{tp+fn},F1=2\frac{RP}{R+P}$$

To imitate this manual process of stimulation adjustment, it is intended that stimulation levels are automatically calibrated by the model based on the previous stimulation level predicted by the model. The amount of pulse amplitude can be gradually increased by 1 mA to reach the maximum level (0, 5, 10, 15, 20 mA for levels 0, 1, 2, 3, 4, respectively). For the stimulation duration, EMS can be continued until RMS_x_ and RMS_y_ are reduced by at least 2.5 times those recorded at the start, indicating a significant tremor reduction.

## Results

### Patients Demographic and Characteristics

The demographic and baseline clinical characteristics are summarized in Table 3. All 20 PD patients were of the tremor predominant subtype, which was confirmed by kinematic studies for a classic type 1 Parkinsonian tremor according to the consensus statement (Deuschl et al., 1998). From Table 4, the reduction of the UPDRS score during stimulation, the reduction of the UPDRS tremor score during stimulation and the UPDRS tremor score for the most affected hand was greater than before stimulation (*p* < 0.001, each) and a consistent reduction of RMS in the x-, y-, and z-axis was achieved (*p* < 0.001, *p* = 0.001, and *p* < 0.001, respectively). However, there was no statistical difference in tremor frequency between the before-stimulation and the during-stimulation measurements in all axes. Mean maximum pulse amplitude was 9.45 (SD ± 4.29) mA (min–max: 3–17, average pulse amplitude was 6.25 (SD ± 2.84) and mean stimulation period was 440.7 (SD ± 560.82) seconds (min–max: 42–2382). During EMS, the duration of tremor reduction during stimulation lasted for 200.35 (SD ± 170.21) seconds (min–max: 42–596) and the duration of continuing tremor reduction after the withdrawal of EMS until the tremor re-emerged to the pre-stimulation level was 129.55 (SD ± 226.7) seconds (min–max: 0–1032). The tremor reset time was 329.90 (SD ± 340.91) seconds (min–max: 73–1628). None of the subjects reported any serious adverse events, including numbness, burning sensation, or fatigue occurring at the stimulation sites during the examination or at the 1-month follow-up visit.

**TABLE 3:** A demographic study of all patients participated in this study.

**Items**	**Description (*N* = 20)**	**Min–Max**
Age (year ± SD)	63.40 ± 9.91	51–85
Hoehn and Yahr (Score ± SD)	2.53 ± 0.85	1–4
LED (mg/day ± SD)	761.20 ± 329.69	300–1759
Disease duration (year ± SD)	8.45 ± 3.26	4–18
TMSE (Score ± SD)	26.55 ± 2.80	21–30
**Tremor outcome: before stimulation**		
• Off UPDRS—III	30.90 ± 12.08	14–56
• Off UPDRS—III tremor	9.15 ± 3.63	2–16
• Off UPDRS—III tremor limb	3.65 ± 0.58	2–4
• On UPDRS—III	17.95 ± 7.52	6–33
• On UPDRS—III tremor	5.50 ± 2.31	1–9
• On UPDRS—III tremor limb	2.20 ± 0.52	1–3
• RMS angular velocity X axis	13.19 ± 19.46	0.36–66.46
• RMS angular velocity Y axis	16.04 ± 30.45	0.17–127.65
• RMS angular velocity Z axis	8.25 ± 12.80	0.09–46.59
• Frequency X axis	5.82 ± 1.76	2.32–10.43
• Frequency Y axis	5.29 ± 1.82	2.33–8.91
• Frequency Z axis	5.59 ± 1.60	2.71–8.24
**Tremor outcome: during stimulation**		
• On UPDRS—III during stimulation	16.65 ± 7.53	5–32
• On UPDRS—III tremor during stimulation	4.20 ± 2.35	0–8
• On UPDRS—III tremor limb during stimulation	0.85 ± 0.49	0–2
• RMS angular velocity X axis	5.10 ± 11.11	0.18–50.70
• RMS angular velocity Y axis	6.29 ± 15.31	0.14–68.77
• RMS angular velocity Z axis	3.55 ± 9.39	0.11–43.04
• Frequency X axis	6.47 ± 1.35	3.10–8.96
• Frequency Y axis	5.68 ± 1.22	3.10–7.57
• Frequency Z axis	5.88 ± 1.87	2.32–11.22
**Stimulation parameters**		
• Maximum pulse amplitude (mA ± SD)	9.45 ± 4.29	3–17
• Average pulse amplitude (mA ± SD)	6.25 ± 2.84	2.06–12.95
• Stimulation period (second ± SD)	440.70 ± 560.82	42–2382
• The duration of tremor reduction during stimulation (second ± SD)	200.35 ± 170.21	42–596
• The duration of continuing tremor reduction after the withdrawal of EMS until the tremor re-emerged to the pre-stimulation level (second ± SD)	129.55 ± 226.71	0–1032
• Tremor reset time (second ± SD)	329.90 ± 340.91	73–1628

*UPDRS, Unified Parkinson’s Disease Rating Scale; LED, Levodopa Equivalent Dosage; BMI, Body Mass Index; TMSE, Thai Mental Status Examination; EMS, Electrical muscle stimulation.*

**TABLE 4:** Comparison of tremor parameters between the before-stimulation and during-stimulation measurements.

**Items**	**Before stimulation (Value ± SD)**	**During stimulation (Value ± SD)**	***p*-value**
On UPDRS III	17.95 ± 7.52	16.65 ± 7.53	<0.001
On UPDRS III tremor section	5.50 ± 2.31	4.20 ± 2.35	<0.001
On UPDRS III tremor limb	2.20 ± 0.52	0.85 ± 0.49	<0.001
RMS angular velocity X axis	13.19 ± 19.46	5.10 ± 11.11	<0.001
RMS angular velocity Y axis	16.04 ± 30.45	6.29 ± 15.31	0.001
RMS angular velocity Z axis	8.25 ± 12.80	3.55 ± 9.39	<0.001
Frequency X axis	5.82 ± 1.76	6.47 ± 1.35	0.135
Frequency Y axis	5.29 ± 1.82	5.68 ± 1.22	0.354
Frequency Z axis	5.59 ± 1.60	5.88 ± 1.87	0.872

*UPDRS, Unified Parkinson’s Disease Rating Scale; RMS, Root Mean Square. All statistical analysis was performed by Wilcoxon Signed Rank test.*

### Results of Correlation Analysis

Correlation analysis was performed to evaluate the relationship between stimulation parameters and tremor reduction outcomes according to Table 5. Significant correlations between tremor reset time and the following stimulation parameters were identified: max pulse amplitude (*r* = 0.567, *p* = 0.008), average pulse amplitude (*r* = 0.495, *p* = 0.035), stimulation time (*r* = 0.586, *p* = 0.007), and duration of tremor reduction during stimulation (*r* = 0.773, *p* < 0.001). In addition, significant correlations between tremor reset time and the following tremor reduction outcomes were identified: delta UPDRS tremor limb (*r* = 0.505, *p* = 0.023), delta RMS_x_ (*r* = 0.456, *p* = 0.043), and delta RMS_y_ (*r* = 0.459, *p* = 0.042). Delta is the difference of outcome between before and during stimulation. Furthermore, significant correlations between average pulse amplitude and the following stimulation parameters were identified: max pulse amplitude (*r* = 0.787, *p* < 0.001), duration of continuing tremor reduction after the withdrawal of EMS until the tremor re-emerged to the pre-stimulation level (*r* = 0.474, *p* = 0.035), and tremor reset time (*r* = 0.495, *p* = 0.027). In addition, significant correlations between average pulse amplitude and the following tremor reduction outcomes were identified: delta RMS_x_ (*r* = 0.686, *p* = 0.001), delta RMS_y_ (*r* = 0.789, *p* < 0.001), and delta RMS_z_ (*r* = 0.746, *p* < 0.001). Finally, significant correlations between stimulation and the following stimulation parameters were identified: max pulse amplitude (*r* = 0.552, *p* = 0.012), duration of tremor reduction during stimulation (*r* = 0.866, *p* < 0.001), and tremor reset time (*r* = 0.586, *p* = 0.007).

**TABLE 5:** Correlation of stimulation parameters and tremor reduction outcomes.

**Parameters**	**Stimulation parameters**		**Tremor reduction outcome**	
	**Parameters**	***r***	**Outcome**	***r***
Tremor reset time	Max pulse amplitude	0.567* (*p* = 0.008)	Delta UPDRS tremor limb	0.505* (*p* = 0.023)
	Average pulse amplitude	0.495* (*p* = 0.035)	Delta RMS X	0.456* (*p* = 0.043)
	Stimulation time	0.586* (*p* = 0.007)	Delta RMS Y	0.459* (*p* = 0.042)
	The duration of tremor reduction during stimulation	0.733* (*p* > 0.001)		

Average pulse amplitude	Max pulse amplitude	0.787* (*p* > 0.001)	Delta RMS_X_	0.686* (*p* = 0.001)
	The duration of continuing tremor reduction after the withdrawal of EMS until the tremor re-emerged to the pre-stimulation level	0.474* (*p* = 0.035)	Delta RMS_y_	0.789* (*p* > 0.001)
	Tremor reset time	0.495* (*p* = 0.027)	Delta RMS_z_	0.746* (*p* > 0.001)

Stimulation time	Max pulse amplitude	0.552* (*p* = 0.012)		
	The duration of tremor reduction during stimulation	0.866* (*p* > 0.001)		
	Tremor reset time	0.586* (*p* = 0.007)		

All correlation analysis was performed by Spearman Rho’s correlation; Statistically significant was determined with *p < 0.05; delta: the difference of outcome between before and during stimulation; RMS_x_, RMS Gyroscope axis X; RMS_y_, RMS Gyroscope axis Y; RMS_z_, RMS Gyroscope axis Z.

### Results of the Multiple Linear Regression Analysis

An additional multiple linear regression analysis with a stepwise method was carried out to predict factors that would yield the longest period of tremor reset time. After including multiple parameters to the stepwise model, including; average pulse amplitude, max pulse amplitude, stimulation time, LED, Hoehn and Yahr stage, Age, disease duration, TMSE score, UPDRS III—off period, UPDRS III tremor—off period, UPDRS III tremor limb– off period, UPDRS III—on period, UPDRS III tremor—on period, and UPDRS III tremor limb– on period. We found the average pulse amplitude and the stimulation time were predictive factors for the tremor reset time in a mathematic model: D = −98.336+ 48.559 (E) + 0.282 (A); where “D” represents tremor reset time (seconds), “E” represents the average pulse amplitude (mA), and “A” represents stimulation time (seconds). In this model, the multiple correlation coefficient was 0.712, while the coefficient of the determinant was 50.7%. Based on the conclusions provided by the model, a higher average pulse amplitude and a greater stimulation time results in a longer tremor reset time.

### Results of Automatic Pulse Amplitude Prediction Models

We aimed to find the most effective algorithm to identify which tremor patterns should be stimulated and which pulse amplitude should be provided to obtain the longest tremor reset time. Five machine learning techniques: Logistic Regression (LR), Random Forest (RF), SVM, NN, and LSTM, were evaluated based on threefold cross validation. There were two feature sets in the experiment: (1) only gyroscope signals (3 inputs: RMS_x_, RMS_y_, RMS_z_) and (2) gyroscope signals with the previous stimulation level (4 inputs: RMS_x_, RMS_y_, RMS_z,_ I_t–1_); thus, the same procedures were employed similarly on both feature sets. To obtain the best parameters, the grid search was employed on the traditional machine learning algorithms (RF, SVM, and NN) on both feature sets, except for LR. For LR, we did not perform the grid search with this model since there are only four maximum inputs (RMS_x_, RMS_y_, RMS_z,_ I_t–1_), so we decided to use all of them to avoid underfitting the model. Table 6 shows parameters’ choices employed by the grid search for RF, SVM, and NN on both feature sets. For LSTM, we have compared different network’s settings manually and chosen the best one, e.g., the number of hidden units, the number of layers, etc. The chosen model of LSTM on both feature sets composed of two layers with 100 hidden units followed by a dense layer with softmax activation. The optimizer was “adam” with the learning rate of 0.001 and the categorical cross-entropy loss. In addition, a feature normalization (StandardScaler in the scikit-learn library) was performed as a data preprocessing for SVM, NN, and LSTM.

**TABLE 6:** Choices of parameters used in the grid search for RF, SVM, and NN on both feature sets.

**Parameters**	**Range of parameters**	**The best parameters**	
		**Feature Set: Only gyroscope signals**	**Feature Set: Gyroscope signals with previous stimulation**
**RF (RandomForestClassifier)**			
The number of trees *(n_estimators)*	[50, 100, 200, 300, 400, 500]	200	100
The bootstrap option *(bootstrap)*	[True, False]	True	True
The criterion *(criterion)*	[“gini,” “entropy”]	“gini”	“gini”
The minimum number of samples *(min_samples_leaf)*	[2, 5, 10, 20, 30]	5	2
**SVM (SVC)**			
C *(C)*	[0.5, 1, 2]	2	1
Kernel *(kernel)*	[“linear,” “poly,” “rbf,” “sigmoid”]	“rbf”	“rbf”
**NN (MLPClassifier)**			
The size of hidden layers *(hidden_layer_sizes)*	[(50), (100), (200), (50, 50), (100, 100), (200, 200)]	(100, 100)	(100, 100)
The activation function *(activation)*	[“tanh,” “relu”]	“relu”	“relu”
The optimizer *(solver)*	[“lbfgs,” “sgd,” “adam”]	“adam”	“adam”

Tables 7, 8 show the results in terms of F1 and accuracy, respectively. The results demonstrated that LSTM was the most effective model with F1 = 0.736 and accuracy = 0.865. The success of LSTM is probably found in its time dependency mechanism, so is designed to capture the variation of patient’s movement patterns over time. Also, the use of previous stimulation levels (I_t–1_) played an important role in improving model performance, almost doubling performance on all models and in the most suitable model, LSTM, it improved the performance 149.51% on F1 and 73.87% on accuracy. Table 9 shows an accuracy per class for the most suitable model. It achieved more than 80% accuracy on most classes, however, it could not capture the data in Class 4 due to a scarcity issue for this class, giving only 0.67% as shown in Table 2.

**TABLE 7:** Performance in terms of “macro-F1” comparison between Logistic Regression, Random Forest, NN, and SVM with two feature sets.

**Model**	**Only gyroscope (Value ± SD)**	**Gyroscope with previous stimulus (Value ± SD)**	**Improvement (%)**
Logistic regression	0.211 ± 0.121	0.317 ± 0.144	50.47%
Random forest	0.261 ± 0.110	0.734 ± 0.274	180.85%
SVM	0.261 ± 0.128	0.436 ± 0.183	67.25%
NN	**0.296 ± 0.096**	0.683 ± 0.264	130.56%
LSTM	0.295 ± 0.090	**0.736 ± 0.282**	149.51%

*The last column shows a percentage of improvement by adding the previous stimulation level (I_t–1_). Boldface refers to the most effective in each category in the first two column. SVM, support vector machine; NN, Neural Network; LSTM, long-short-term-memory.*

**TABLE 8:** Performance in terms of “accuracy” comparison between Logistic Regression, Random Forest, NN, and SVM with two feature sets.

**Model**	**Only gyroscope (Value ± SD)**	**Gyroscope with previous stimulus (Value ± SD)**	**Improvement (%)**
Logistic regression	0.292 ± 0.285	0.472 ± 0.363	61.62%
Random forest	0.431 ± 0.292	0.846 ± 0.292	68.18%
SVM	0.404 ± 0.297	0.680 ± 0.295	96.39%
**NN**	**0.513 ± 0.252**	0.840 ± 0.254	63.84%
LSTM	0.497 ± 0.212	**0.865 ± 0.259**	73.87%

*The last column shows a percentage of improvement by adding the previous stimulation level (I_t–1_). Boldface refers to the most effective in each category on the first two columns. SVM, support vector machine; NN, Neural Network; LSTM, long-short-term-memory.*

**TABLE 9:** Accuracy per class of the most suitable model in this study (LSTM).

**Class**	**0**	**1**	**2**	**3**	**4**
Accuracy	99.76%	87.43%	73.92%	74.17%	0.0%

*The most suitable model with the highest accuracy is the combination of LSTM analysis using gyroscope data and the previous stimulation level.*

## Discussion

In this study, we evaluated the efficacy of EMS in the suppression of classic parkinsonian tremor in 20 PD patients using a Parkinson’s glove. The results of tremor reduction were significant and consistent with prior findings (Gallego et al., 2013; Dosen et al., 2015). However, no previous studies have reported quantitative measurements of tremor attenuation after stimulation to allow further comparisons (Jitkritsadakul et al., 2015, 2017). Here, we quantified the duration of the tremor from initiation of EMS to the point after EMS withdrawal when tremor returned to pre-stimulation levels. We found that the mean maximum pulse amplitude applied was 9.45 (SD ± 4.29) mA and the mean stimulation period was 440.7 (SD ± 560.82) seconds and the mean total tremor reset time was 329.90 (SD ± 340.91) seconds. The multiple linear regression model showed that a longest tremor reset time could be obtained by increasing pulse amplitude and increasing the stimulation time. These findings further improve our knowledge on the peripheral mechanism of tremor and the effect of stimulation in the suppression of resting tremor. EMS may act as strong stimuli that can transiently modify tremors, even if the tremor originally occurred from a central origin (Rack and Ross, 1986; Britton et al., 1993; Hallett, 1998; Deuschl et al., 2001).

### The Tremor Reset Mechanism

Tremor in PD is generated by the interaction between central and peripheral mechanism (Supplementary Figure 2; Hallett, 1998; Deuschl et al., 2000, 2001; McAuley and Marsden, 2000). Early findings on resting tremors in PD suggest they can be reset by an alteration in mechanical conditions at the periphery, including externally imposed movement at a joint or electrical stimulation of nerves and muscles (Mones and Weiss, 1969; Bathien et al., 1980; Lee and Stein, 1981; Britton et al., 1992, 1993). The potential resetting of parkinsonian tremors by modulation of peripheral reflex mechanisms using a short-duration, supramaximal peripheral nerve stimulation was first explored by Mones and Weiss (1969). A spontaneous tremor in the extensor digitorum muscle changed dramatically after the ipsilateral stimulation of median or ulnar nerves with a single 0.5 ms duration, square wave, and supramaximal stimulation and the interval of the post-stimulation tremor bursts decreased when compared with control values. In addition, there was a change in the predicted tremor pattern of the post-stimulation tremor bursts, which were out of phase with the pre-stimulation tremor bursts (Mones and Weiss, 1969). The shortened interval increased gradually on subsequent spontaneous tremor bursts and returned to the control interval value in 750–1,000 ms after nerve stimulation (Mones and Weiss, 1969). Therefore, the pattern of parkinsonian tremor appeared to be “reset” by the peripheral nerve stimulation. After recording the spontaneous tremor of the extensor indicis muscle after a single 1 ms duration square wave and supramaximal stimulation of the radial nerve, the interval of the next post-stimulation tremor burst was slightly delayed, especially if the stimulation occurred late in the tremor cycle (Bathien et al., 1980). This findings needed to be investigated further to see if there is potential for longer periods of supramaximal stimulation to produce a greater interval for subsequent post-stimulation tremors. Our previous study proposed that resting tremor appeared to be reset after increasing the duration of stimulation (Jitkritsadakul et al., 2015, 2017). After applying EMS using a 10–30-s duration square-wave stimulation, some patients had continuing tremor reduction where a longer duration of stimulation produced a greater duration of reset time (Jitkritsadakul et al., 2015, 2017). The temporary suppression of parkinsonian tremors from EMS may occur via the stretch reflex in muscle fibers (Jitkritsadakul et al., 2015) or by the inhibitory effect of the muscle contraction with Ib fiber rather than Ia fiber (Bathien et al., 1980). Alternatively, EMS may be able to modulate Renshaw cells (Pratt and Jordan, 1987; Alvarez et al., 2013), which create a negative feedback mechanism in the spinal cord and possibly led to tremor suppression (Jitkritsadakul et al., 2015).

The Parkinson’s glove is an innovative device with the potential to become an alternative treatment option for patients with medically refractory tremor. This current study is the first that we are aware of that has described the effect of EMS on tremor reduction, and propose a model for calculating tremor reset times. We also identified a machine learning model to predict the pulse amplitude required to achieve the greatest tremor reset time. Using the machine learning model will allow physicians employing this device to identify the most suitable tremor paradigm to suppress and the optimal stimulation protocols to use. However, our study contains certain limitations. Firstly, as this open-label, quasi-experimental study was conducted in a small single group of patients collected from a single PD center in Thailand, it may retain selection and sampling bias that comes with a single group study. However, our study is the ability to recruit the homogeneous PD population with the homogeneous data by selecting a group of PD patients with Class I parkinsonian tremor as confirmed by quantitative tremor analysis, therefore, the characteristic of PD tremor in this study was homogeneous and well represented. Secondly, due to the IRB granted us to permission this treatment as an adjunctive treatment during “on” period when patients were optimized on oral medications, therefore, during stimulation section was conducted during “on” period only. Therefore, this study gave the potential benefit of EMS as an adjunctive treatment for the specific class I parkinsonian tremor. However, for future direction, EMS should be further explored as a possible therapeutic intervention for the different state of tremors in PD (such as “off” period) or the potential usage in other tremor syndromes. Thirdly, the data set that was used to train the model to identify significant tremor reduction was judged by the criteria of significant tremor reduction only. As the impact of EMS on tremor reduction was a transient effect and could not be explained by pre-existing severity of disease or tremor, there are no results to confirm the benefit of EMS in a long-term study. Fourthly, as a longer duration of electrical stimulation is more painful than a shorter duration (Walsh et al., 1998; Tamura et al., 2013), we kept the maximum pulse stimulation below the patients’ pain thresholds, delivered as short periods of stimulation to avoid potential side effects of EMS, and, therefore, it is not clear if longer durations of stimulation would support tremor reduction in a long term. Though, the issue of tolerance was not investigated in this study, it has been documented in PD patients with thalamic stimulation (Hariz et al., 1999). Finally, using collected data from the physician’s process, we demonstrated that the model could predict a suitable stimulation level based on the current tremor states and the previous stimulation level. However, as the testing phase was conducted using the previous stimulation levels from the collected data, not from the model prediction, the model will require further development before it can be deployed in a real-life patient scenario.

Going forward, it is our aim to use this model to automatically stimulate PD patients to achieve optimal tremor suppression. In the manual process, a physician provides a suitable stimulation level based on the previous pulse amplitude level until a patient shows a significant tremor reduction. To imitate this process, the model is being developed so that the stimulation level will be automatically based on the previous stimulation level predicted by the model. Then, in future work, we can evaluate whether or not the model can achieve tremor reset times similar to that of manual stimulation.

## Conclusion

Our study provides evidence of the efficacy of EMS in transient tremor reduction among PD patients with medically intractable rest tremor, providing calculations for a model to predict the tremor reset time and evaluation of the best machine-learning model for automatic prediction of the suitable pulse amplitude for stimulation. Greater pulse amplitude and stimulation periods may result in longer tremor reset times. From the machine learning model evaluation, LSTM was identified as the most effective model for the prediction of stimulation levels, and, in future, could be used to automatically adjust stimulation levels, replicating the manual process periods of tremor reset time. Our study also provides more insight into the role of peripheral mechanisms in the origin of parkinsonian tremor. Targeting peripheral mechanisms with strong stimuli may not be able to stop, but could modulate tremor amplitude, even if it is mainly driven from a central origin. The efficacy of EMS should be explored in other tremor syndromes.

## Data Availability Statement

The raw data supporting the conclusions of this article will be made available by the authors, without undue reservation.

## Ethics Statement

The studies involving human participants were reviewed and approved by the Institutional Review Board, Faculty of Medicine, Chulalongkorn University (IRB 483/57). The patients/participants provided their written informed consent to participate in this study.

## Author Contributions

OP and RB designed the research and drafted the work. OP, PV, IW, and CA performed the experiments. OP and PV analyzed the data. All authors contributed to the article and approved the submitted version.

## Conflict of Interest

The authors declare that the research was conducted in the absence of any commercial or financial relationships that could be construed as a potential conflict of interest.

## Publisher’s Note

All claims expressed in this article are solely those of the authors and do not necessarily represent those of their affiliated organizations, or those of the publisher, the editors and the reviewers. Any product that may be evaluated in this article, or claim that may be made by its manufacturer, is not guaranteed or endorsed by the publisher.

## Footnotes

1
www.chulapd.org

## Supplementary Material

Supplementary Figure 1 The CONSORT flowchart provides a complete detailed flowchart of this single-arm study.

Supplementary Figure 2 The complex interaction between central and peripheral (local mechanical-reflex) mechanisms for tremorogenesis. Representation of the origination of PD tremor that develops from the complex interaction between central oscillators and peripheral mechanisms. Bold lines represent the tremor control commanded from central oscillators to peripheral mechanisms Dashed lines represent feedback loops from peripheral mechanisms back to central control oscillators.

Supplementary Material 1 Translation of protocol synopsis No. 483/57 (NCT02370108).

Supplementary Material 2 A TREND checklist.

Supplementary Material 3 Sample size calculation.
