# Supplementary figures and images for: Using Machine Learning for Predicting the Best Outcomes With Electrical Muscle Stimulation for Tremors in Parkinson’s Disease
Source: Front Aging Neurosci. 2021 Sep 10;13:727654. doi: 10.3389/fnagi.2021.727654 (PMC8461308; doi:10.3389/fnagi.2021.727654)

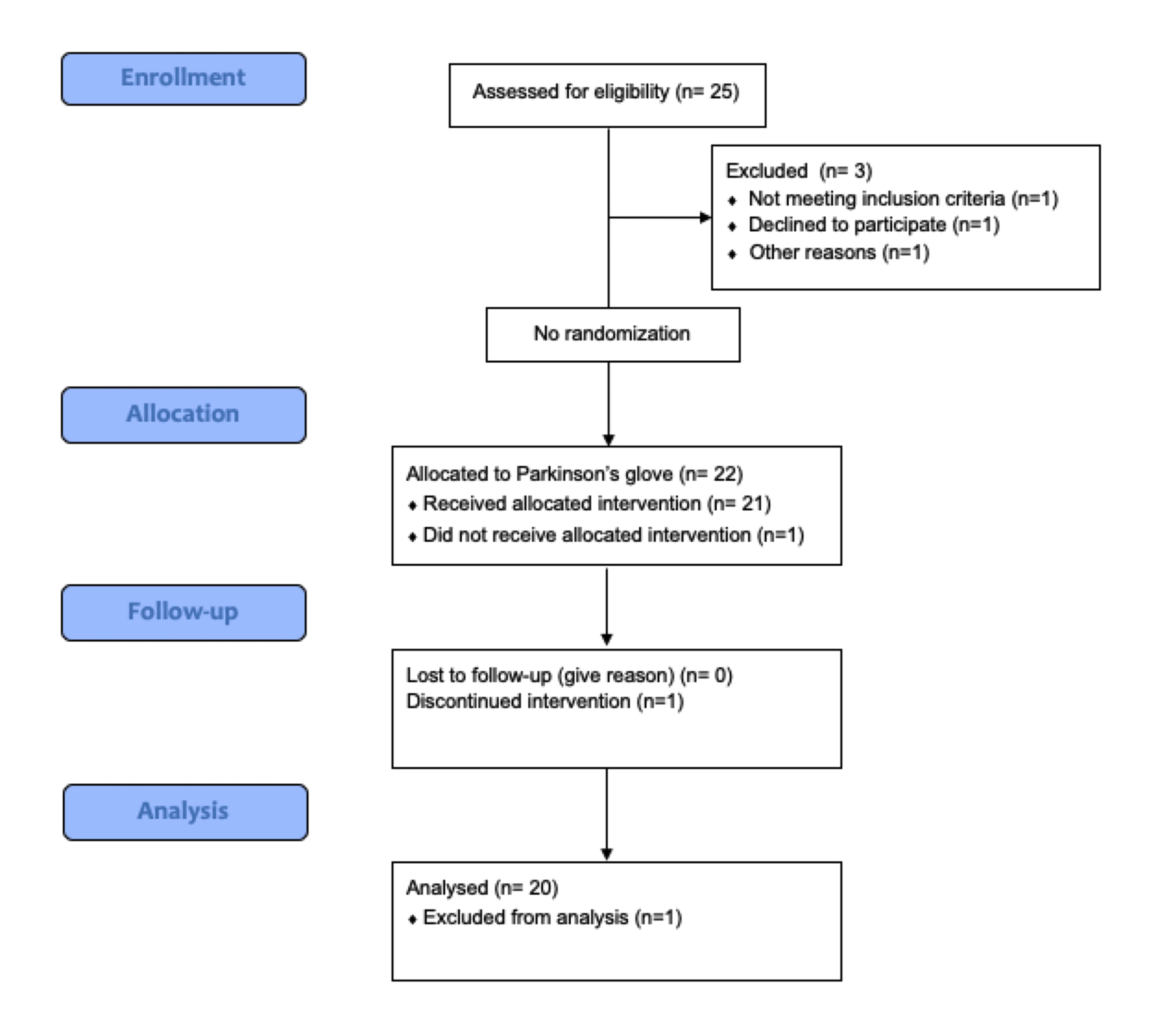

Supplement: Supplementary Figure 1 — The CONSORT flowchart provides a complete detailed flowchart of this single-arm study. [file Image_1.JPEG]

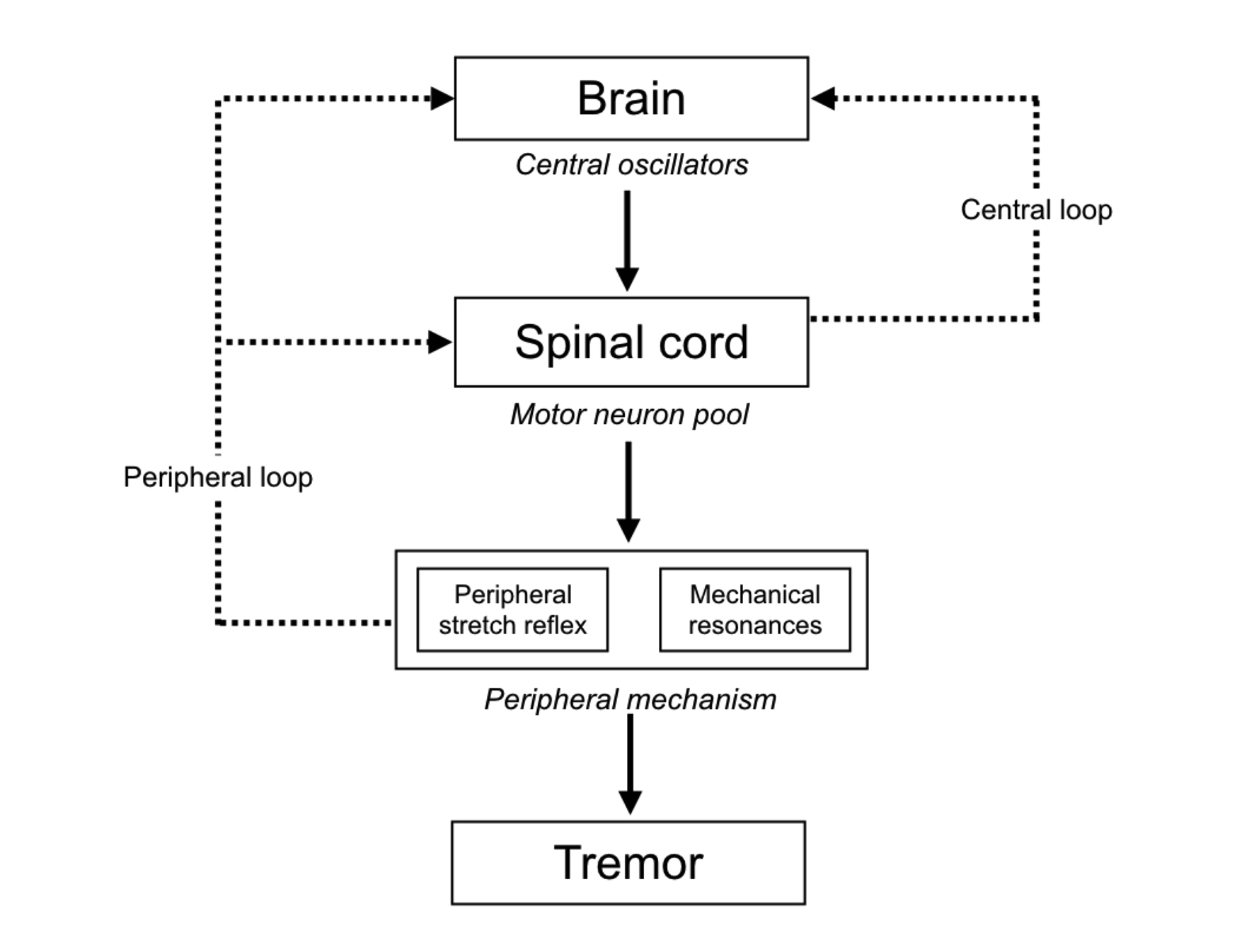

Supplement: Supplementary Figure 2 — The complex interaction between central and peripheral (local mechanical-reflex) mechanisms for tremorogenesis. Representation of the origination of PD tremor that develops from the complex interaction between central oscillators and peripheral mechanisms. Bold lines represent the tremor control commanded from central oscillators to peripheral mechanisms Dashed lines represent feedback loops from peripheral mechanisms back to central control oscillators. [file Image_2.JPEG]
