# Supplementary material for: Using Machine Learning for Predicting the Best Outcomes With Electrical Muscle Stimulation for Tremors in Parkinson’s Disease
Source: Front Aging Neurosci. 2021 Sep 10;13:727654. doi: 10.3389/fnagi.2021.727654 (PMC8461308; doi:10.3389/fnagi.2021.727654)
Supplement: Supplementary Material 1 — Translation of protocol synopsis No. 483/57 (NCT02370108). [file Data_Sheet_1.doc]

**Protocol Synopsis**

1. Title of study

A Study of Rest Tremor Suppression by Using Electrical Muscle Stimulation

2. Principal Investigator

Dr. Onanong Phokaewvarangkul (Jitkritsadakul)

3. Study centers & Address

- Chulalongkorn Center of Excellence for Parkinson’s disease and Related Movement Disorders, Chulalongkorn Hospital, 1873, Rama 4 Road, Pathumwan, Bangkok 10330
- Geriatric Clinic, Chulalongkorn Hospital, 1873, Rama 4 Road, Pathumwan, Bangkok 10330
- Internal Medicine Clinic, Chulalongkorn Hospital, 1873, Rama 4 Road, Pathumwan, Bangkok 10330

4. Study period

2 years

5. Objectives

- To study the effect of electrical muscle stimulation on reducing resting tremors in Parkinson's disease patients.
- To compare the effect of electrical muscle stimulation on reducing resting tremors between Parkinson's disease patients and other tremors.
- To compare the results of tremor parameters at rest between 2 tremor analysis devices (standard tremor analysis device and our center developed tremor analysis device)

6. Study design

Experimental (a single-arm, non-randomized controlled) Study

7. Study population and Sample size

The total sample size was 60 participants (divided into 30 patients with Parkinson's disease and 30 patients with others tremor syndromes). Parkinson's patients receiving treatment at Chulalongkorn Center of Excellence for Parkinson’s disease and Related Movement Disorders, Chulalongkorn Hospital, who met the inclusion criteria and did not meet the exclusion criteria. Patients with various tremor syndromes who were at the same age and gender as Parkinson's patients and were recruited from the Internal Medicine Clinic and the Geriatric Clinic of Chulalongkorn Hospital.

*Inclusion criteria for PD patients*

- Adults ≥ 18 years old
- The patient has Thai nationality
- Patients with Parkinson’s disease diagnosed according to the UKPDSBB criteria with predominantly feature of rest tremors that are intractable to medically treatment.
- Patient express their participation in the study and can sign their consent to participate in the study.

*Exclusion criteria for PD patients*

- Patients with the following diseases; cardiac arrhythmia, Epilepsy or patients at risk of seizures, such as severe abnormal of their electrolytes, patients with liver failure or kidney failure, patients with brain pathologies that may develop seizures, such as those with brain tumors, encephalitis patients and patients who have previously suffered a stroke, patient with abrasion wound or injuries on their wrist, patients with a history of hand surgery and implanted screws or wires in the hand area, and patients with a pace maker or patients with deep brain stimulation
- Patients who cannot avoid the medication that may potentiate or attenuate tremors for at least 2 weeks before participating in a research project including, sleeping pills, thyroid drugs, antihistamines, mucus-reducing drugs, alcohol, narcotics, stimulants, and coffee.

*Inclusion criteria for patients with various tremor syndrome*

- Adults ≥ 18 years old
- The patients have Thai nationality
- Patients present with various tremor syndrome
- Patients were of the same age and gender as PD patients
- Patients express their participation in the study and can sign their consent to participate in the study.

*Exclusion criteria for patients with various tremor syndrome*

- Patients with the following diseases; cardiac arrhythmia, Epilepsy or patients at risk of seizures, such as severe abnormal of their electrolytes, patients with liver failure or kidney failure, patients with brain pathologies that may develop seizures, such as those with brain tumors, encephalitis patients and patients who have previously suffered a stroke, patient with abrasion wound or injuries on their wrist, patients with a history of hand surgery and implanted screws or wires in the hand area, and patients with a pace maker or patients with deep brain stimulation
- Patients who cannot avoid the medication that may potentiate or attenuate tremors for at least 2 weeks before participating in a research project including, sleeping pills, thyroid drugs, antihistamines, mucus-reducing drugs, alcohol, narcotics, stimulants, and coffee.

**Method and sampling technique**

Patients who participate in this study will be explained for details of the whole research and inform the research project requirements as follows

- Patients will be able to read the research literature and be explained in detail the research project including, research objectives, research process, research benefits, research methods, withdrawing process from this research, and how to keep confidential for patient information, etc. The patient can ask all questions related to this study and have sufficient time to decide for participation. A written consent to participate in research will be signed before participate in the research
- Demographic information of both groups of patients will be recorded including, gender, age, occupation, education level, underlying disease, disease duration, current medications, history of alcohol use, smoking, and substance use, and family history.
- Both groups of patients who participated in the study will be asked for their medical history, collected their vital signs, and conducted a neurological examination. In addition, patients with Parkinson's disease will be diagnosed according to United Kingdom Parkinson's Disease Society Brain Bank's clinical criteria for the diagnosis of probable Parkinson's disease (UKPDSBB) criteria.
- Participants were required to discontinue any drugs or substances that could influence to tremors for at least 2 weeks, including sleeping pills, thyroid drugs, antihistamines, mucus-reducing drugs, alcohol, narcotics, stimulants, and coffee for 2 weeks prior to participating in the research project. Patients will be discussed for possible risks that may arise during the drug discontinuation, such as insomnia, and anxious. However, to reduce the symptoms, patients will be given self-care instructions such as sleep hygiene, sleep adjustments, and how to reduce insomnia without using oral medication, including using topical drugs, etc., and on the day of the examination, the patient must not be in a sleep-deprived state and do not eat caffeinated drinks before the examination.
- All study participants will be assessed using the Thai Mini Mental Status Examination.
- Patients diagnosed with Parkinson's disease will assessed with a standard rating scale, called the Unified Parkinson's Disease Rating Scale (UPDRS) during oral medication period in order to assess for medication-refractory tremors.
- All study participants will be tested with tremor analysis device and stimulation their hand muscles with a surface electrode of an electrical muscle stimulation (thenar muscle & 1st-2nd interosseous muscle). The stimulation will be used with a low, safety range, current (up to 20 mA) until had tetanic contractions without pain (Frequency between 30-50 Hz). During the examination period, tremor analysis devices will be used to monitor or patient’s tremor (standard tremor analysis device and our center developed tremor analysis device).
- All study participants were collected the tremor data with tremor parameters ​are as follows: peak magnitude, RMS of angular velocity, frequency, Q wave, etc.
- The test will conduct approximately 30 minutes, after the test if the patient had no side effect they can be discharged.

**Data collection**

- Demographic information of both groups of patients will be recorded including, gender, age, occupation, education level, underlying disease, disease duration, current medications, history of alcohol use, smoking, and substance use, and family history.
- Participants were classified into two groups: the Parkinson's disease will be diagnosed using The United Kingdom Parkinson's Disease Society, Brain Bank's clinical criteria for the diagnosis of probable Parkinson's disease and the control group.
- Participants were required to discontinue any drugs or substances that could influence to tremors for at least 2 weeks prior to participating in the research project.
- All study participants will be assessed using the Thai Mini Mental Status Examination.
- All study participants were collected the tremor data with tremor parameters ​are as follows: peak magnitude, RMS of angular velocity, frequency, Q wave.

**Disclosure of information that identifies the patient**

Patient identification information will be kept confidential. The identity of the patient is never disclosed. For data analysis, the code will be used for each patient. In publication of research results or academic presentations, overall research results are presented. The identity of the patient is never disclosed. If necessary, personally identifiable information is presented to the patient. Only written consent from the patient is required.

8. Investigational product, dosage & route of administration

Electrical muscle stimulation in the frequency range of 30-50 Hz and pulse amplitude less than 20 mA

9. Comparator, dosage & mode of administration

None

10. Duration of participation for each volunteer

All PD patients will be applied a questionnaire and taken one test on the day of their visit in the doctor’s office. Other tremor patients will be applied a questionnaire and taken one test on the day of their visit in the doctor’s office.

11. Duration of study

2 years

12. Statistical method

All data will be analyzed using SPSS program, mainly with non-parameter statistics such as chi-square, Mann-Whitney U test, Wilcoxon Sign Rank test, etc. The statistic is calculated at <0.05 confidence.

13. Ethical Consideration

The study will follow the principles of research ethics in 3 points:

1. Principle of Respect for person by providing complete information about research until the volunteers fully understand and independently decide to give consent to participate in research. The patient's decision to participate in the study or not will not have any effect for treating patients.
2. Principles of Benefit Not causing harm (Benefience / Non-maleficence) Patients will be able to benefit from tests such as tremor evaluation. The device used to measure the tremor analysis device that causes no painful or harmless program. Devices used to stimulate electric current are medical devices used in physical therapy with a low current that cause no pain. The study will also focus on patient data privacy.
3. The principle of justice (Justice) In this study, the criteria for in and out are clearly defined and diversify risks and equally benefit by using random method to enter study group
