# Supplementary material for: Using Machine Learning for Predicting the Best Outcomes With Electrical Muscle Stimulation for Tremors in Parkinson’s Disease
Source: Front Aging Neurosci. 2021 Sep 10;13:727654. doi: 10.3389/fnagi.2021.727654 (PMC8461308; doi:10.3389/fnagi.2021.727654)
Supplement: Supplementary Material 3 — Sample size calculation. [file Data_Sheet_3.docx]

**Supplementary Material 3:** Sample size calculation

We calculated the sample size in this study based on the formula for the paired data as follows:

$$N =\frac{{\left( Z_{\alpha/2} \right.+\left. Z_{\beta} \right)}^{2}\left( \sigma\right)^{2}}{d^{2}}$$

Where: N = sample size

$\sigma$ = standard deviation of the within-pair difference

d = clinically meaningful difference

Z**_β_** = corresponds to power

Z**_α/2_** = corresponds to two-tailed significance level

The sample size was calculated based on the RMS of the angular velocity between before and during stimulation as the main outcome. The RMS angular velocity before stimulation was 51.60 ± 51.07 degree/s and was 31.19 ± 35.55 degree/s during stimulation. Therefore, we need at least 6.07 or 7 patients to compare tremor parameters before and during stimulation.

**Reference**

1. Jitkritsadakul O, Thanawattano C, Anan C, Bhidayasiri R. Exploring the effect of electrical muscle stimulation as a novel treatment of intractable tremor in Parkinson's disease. J Neurol Sci 2015;358:146-52.

2. Devane D, Begley C, Clarke M. How many do I need? Basic principles of sample size estimation. J Adv Nurs. 2004; **47**(3): 297–302.
